# A Mobility-Aware Adaptive Duty Cycling Mechanism for Tracking Objects during Tunnel Excavation

**DOI:** 10.3390/s17030435

**Published:** 2017-02-23

**Authors:** Taesik Kim, Hong Min, Jinman Jung

**Affiliations:** 1 Department of Civil Engineering, Hongik University, Seoul 04066, Korea; taesik.kim@hongik.ac.kr; 2 School of Computer and Information Engineering, Hoseo University, Asan 31499, Korea; hmin@hoseo.edu; 3 Department of Information and Communication Engineering, Hannam University, Daejeon 34430, Korea

**Keywords:** tunnel excavation, WSNs, adaptive duty cycle, mobility

## Abstract

Tunnel construction workers face many dangers while working under dark conditions, with difficult access and egress, and many potential hazards. To enhance safety at tunnel construction sites, low latency tracking of mobile objects (e.g., heavy-duty equipment) and construction workers is critical for managing the dangerous construction environment. Wireless Sensor Networks (WSNs) are the basis for a widely used technology for monitoring the environment because of their energy-efficiency and scalability. However, their use involves an inherent point-to-point delay caused by duty cycling mechanisms that can result in a significant rise in the delivery latency for tracking mobile objects. To overcome this issue, we proposed a mobility-aware adaptive duty cycling mechanism for the WSNs based on object mobility. For the evaluation, we tested this mechanism for mobile object tracking at a tunnel excavation site. The evaluation results showed that the proposed mechanism could track mobile objects with low latency while they were moving, and could reduce energy consumption by increasing sleep time while the objects were immobile.

## 1. Introduction

There are a variety of types of civil engineering structures such as bridges, dams, embankments, roads, canals, slopes, and tunnels. In Korea, 225 accidents occurred at civil engineering construction sites from 2001 to 2015, in which 173 workers died, according to the construction safety management information system operated by the Korean Ministry of Land, Infrastructure, and Transport [1]. At tunnel construction sites, 30 workers died in 56 work accidents during the interval mentioned, and the cost of the damage was approximately three million dollars. This was the greatest damage among all civil construction accidents. The tunnel construction sites remain dangerous.

A tunnel is an artificial underground passage that is excavated through soil and rock. There are many tunneling methods, but the New Austrian Tunneling Method (NATM) is widely used in engineering practice when constructing tunnels without removing the ground above. Typically, NATM requires various types of heavy-duty equipment and explosives to make a new underground tunnel. As one might expect, the construction workers face many dangers working in the dark, with difficult access and egress, and with many potential hazards [2]. Although efforts to minimize accidents have been made, such as training programs for construction workers, safer tools, and the establishment of strict regulation, a definite answer is still needed to ensure safety.

To enhance safety at tunnel excavation sites, an object tracking system using sensors tagged on heavy-duty equipment and workers might be useful. For example, if a worker got too close to moving heavy-duty equipment, the tracking system could prevent collision accidents by providing warnings to both. In the case of detonation events, all the objects (and personnel) that should be out of the blast area could be checked and confirmed by the tracking system. Note that the sensors are attached to moving objects including the workers, so an electric battery would be an appropriate power source for the sensors. Tunnel construction time varies depending on the tunnel length, but typically takes several years. Hence, to minimize battery replacement while track moving objects over the entire construction time, an adaptive duty cycling mechanism is required for the devices making up the object tracking system.

In this paper, we propose a novel mobility aware adaptive duty cycling mechanism for tracking objects at a tunnel construction site. Unlike existing approaches that focused on adaptation strategies of synchronization frequency, frame length, slot time, and handover to efficiently address the problem of mobility, we concentrate on adaptive duty cycling according to the node mobility, making it suitable for tracking objects with a given energy constraint. Our adaptation strategy aims to enhance network lifetime while reducing response time when objects are mobile. It tunes the duty cycle ratio by adjusting the sleep time depending on the change of Received Signal Strength Indication (RSSI) value. We investigated the energy consumption and latency of this adaptive duty cycling mechanism for various mobility conditions. Our approach was to meet the Quality of Service (QoS) requirements of the energy consumption and latency by considering the mobility conditions in tunnels. Note that GPS signal is typically utilized when developing an object tracking mechanism, but the GPS signal loss frequently occurs in construction sites. The extreme case of this environment is the tunnel construction sites because of its underground environment. Hence, the object tracking mechanism using GPS is not considered in this study.

The rest of this paper is organized as follows: Section 2 is an introduction to the background and related information about duty cycling in WSNs. Section 3 presents the mobility aware adaptive duty cycling mechanism for tracking objects in tunnels. Section 4 is an evaluation of the energy consumption and latency of both adaptive and periodic duty cycling mechanisms. Finally, Section 5 summarizes the work.

## 2. Background and Related Work

### 2.1. Duty Cycling Concept

Human safety is the most important issue and the highest priority in many applications. WSNs can help people protect their life and avoid critical accident by continuous monitoring of moving objects and environments. In Intelligent Transport System (ITS), WSNs that use densely deployed sensors along the road and collect higher spatial resolution of traffic information improve the safety [3]. To use WSNs to reduce cable cost, deployment time, and uncontrolled processes in automation industry [4]. There can be losses and damages to workers and products if the automation equipment is not designed to reduce the risk of uncontrolled or dangerous situations. Struck-by-falling-object accident is the second leading cause of death in construction [5]. WSNs can reduce this accident by providing real-time location tracking and information sharing. In these kinds of applications, saving energy of self-powered sensor nodes is one of the critical issue because it is difficult and time-consuming process to detect the battery problem although they are located where the operator can access them.

Battery-operated wireless devices must have strict power management strategies to prolong node lifetime [6]. The radio interface usually consumes more energy than other parts such as the CPU, memory, and sensors. This radio interface uses as much energy when it is waiting to receive data from other devices (i.e., idle listening) as when it is sending data to other devices [7]. Therefore, a sensor node saves its energy by using a duty cycling mechanism that periodically turns the radio interface on and off. The main goal of duty cycling is to reduce idle listening time [8]. However, it is difficult to turn on the radio interface only when it is necessary [9]. Duty cycling also has some side-effects such as increasing the collision rate, latency in message transmission, and the need for additional control messages [10]. Some duty cycling schemes reduce transmission and reception time windows. These smaller time windows increase the probability of collisions. In the case of multi-hop network, message transmission is delayed because a sender occasionally has to wait for the next hop to wake up. Duty cycling also needs extra control traffic. The most common source of this overhead is synchronization. Achieving a practical balance between the network performance and energy saving is a complex problem, and many duty cycling mechanisms studies have been performed to solve this problem.

### 2.2. Synchronous and Asynchronous Duty Cycling

**Figure 1:**
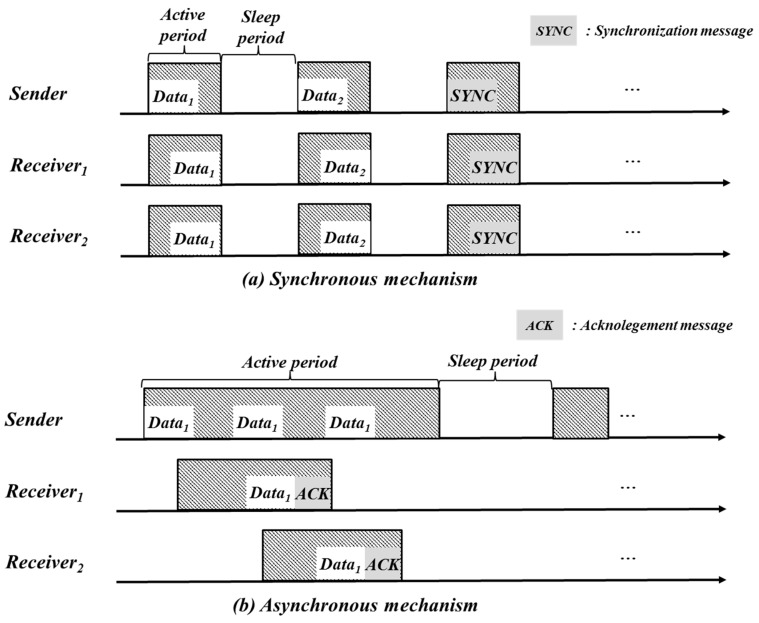
Synchronous and asynchronous duty cycling mechanisms (redrawn from [11]).

Duty cycling mechanisms for static topology are classified into synchronous and asynchronous schemes as shown in Figure 1 [11]. In the case of a synchronous scheme, entire nodes periodically synchronize their clock and transmit data among nodes according to a predetermined schedule. In the case of the asynchronous scheme, nodes do not have to keep a global clock for synchronizing and a sender repeatedly sends the same data to a receiver until the receiver replies with an acknowledgement message after receiving the data. Our duty cycling mechanism is a kind of the asynchronous scheme because objects are moving and a network topology is also changed frequently in tunnel construction site.

For Sensor-MAC (S-MAC) [12], neighboring nodes form groups (i.e., virtual clusters), and synchronize the clock of member nodes within each virtual cluster because it takes high overhead to synchronize all the nodes with one global clock. Each node broadcasts its sleep schedule to the neighbors in the same virtual cluster and records its silent time in a variable called Network Allocation Vector (NAV). If NAV is not zero, the node determines that the wireless media is busy and increases the value of NAV to avoid collision and overhearing.

Timeout-MAC (T-MAC) [13] was proposed to improve S-MAC (with fixed active/sleep duty cycles) by dynamically adjusting the end of active periods. In order to determine an active period, T-MAC defined a threshold time interval (i.e., Time of Arrival or TA). In this system, an active period ends when no data transmission has occurred for time TA. Finding an optimal TA determines the performance of T-MAC because TA indicates the minimal amount of idle listening per frame. T-MAC has a problem called early sleep, which means that a receiver goes to sleep when a sender still has packets for the receiver.

Both S-MAC and T-MAC are synchronous duty cycling mechanisms and not suitable for burst traffic and dynamic network topology. Various asynchronous mechanisms were proposed to support dynamic and mobile networks. Berkeley-MAC (B-MAC) [14] provides an initial idea of an asynchronous mechanism. B-MAC employs an adaptive preamble sampling scheme to reduce duty cycle and minimize idle listening. Every node goes to sleep asynchronously and wakes up periodically to check for channel activity. Whenever a node wakes up, the node turns on the radio and checks for channel activity. If activity is detected, the node powers up and stays awake long enough to complete data reception. If the channel becomes inactive, the node goes back to sleep.

X-MAC [15] employs a short preamble instead of the long preamble used by B-MAC to reduce energy consumption and latency. X-MAC uses two methods, embedding a receiver’s address and using a strobed preamble. The former is to embedded receiver’s address information in the preamble so that non-target receivers go back to sleep shortly. This method can save the energy of non-target receivers but makes broadcasting messages to neighbor nodes difficult. The latter is to allow a target receiver to interrupt the long preamble as soon as the receiver wakes up. The short strobed preamble reduces the time and energy wasted while waiting for complete transmission of the entire preamble.

ContikiMAC [16] was designed to maximize the radio off time. Receiver nodes periodically wake up to listen for packet transmissions from neighbors. If a packet transmission is detected during a wake up, the receiver stays awake to receive the packet. When the packet is completely received, the receiver sends an acknowledgment to the sender. A sender repeatedly sends its packet until the sender receives an acknowledgement from the receiver. ContikiMAC uses the RSSI of the radio transceiver to determine the active state of the channel. A sender only tries to send a packet when RSSI is below a given threshold indicating that the channel is clear to avoid collision. These protocols were designed under the static network topology, but the topology can be changed dynamically in mobile wireless sensor networks.

### 2.3. Adaptive Duty Cycling Based on Mobility

There are many duty cycling schemes considering the mobility of sensor nodes and adjusting their schedule or hands-off request timing adaptively as the speed of the mobility nodes. The mobility-aware MAC protocol for sensor networks (MS-MAC) [17] was proposed to support the mobility of sensor nodes. In MS-MAC, stationary nodes work like S-MAC and mobile nodes work like IEEE802.11 [18]. Each node periodically broadcasts its schedule in a SYNC message to maintain synchronization and measures the RSSI of its neighbors. If there is a change of RSSI from a neighbor, it means that the neighbor or the node is moving and the speed can be estimated. This mobility information is included in the SYNC message and neighbors that receive the message increase the frequency of the synchronization period to maintain connection with the moving node.

**Figure 2:**
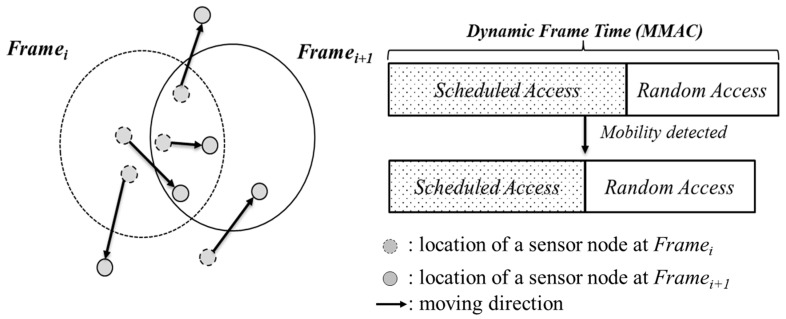
Location estimation and dynamic frame time in MMAC (redrawn from [19]).

Mobility adaptive collision free MAC for mobile sensor networks (MMAC) [19] was based on time division scheduling and designed to be suitable for both high and low mobility environments. At the beginning of each frame, all the nodes predict their position in the next frame and send this estimated location information to their cluster head. The cluster heads that never go to sleep collect the estimated location information of all member nodes and broadcast collected information to their member nodes. Each member node suggests its frame duration based on the collected location information and sends its decision to the cluster head. The cluster head adjusts according to member node requests as shown in Figure 2.

Mobile Cluster MAC (MCMAC) [20] was proposed to optimize for mobile nodes that travel in a cluster (i.e., without inter-cluster mobility). MCMAC classifies sensor nodes into static and mobile clusters. Because the slot assignment method is different for static and mobile clusters, the active period is divided into Static Active Slots (SAS) and Mobile Cluster Slots (MCS). The SAS follows scheduling based communication and the MCS follows contention based communication. All nodes can receive a message during SAS and MCS, but a node in a static cluster sends a message only during the SAS, and a node in a mobile cluster sends a message only during the MCS.

**Figure 3:**
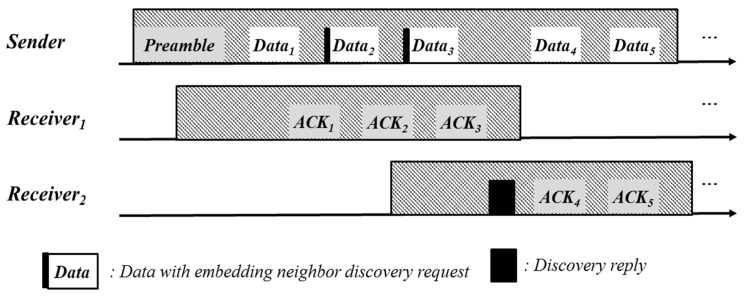
Mobility support in MA-MAC (redrawn from [21]).

Light-weight Mobility-Aware MAC (MA-MAC) [21] for wireless sensor networks was also proposed. MA-MAC extends X-MAC (a contention based scheme) and provides short preambles. MA-MAC detects mobility by using the RSSI of ACK packets and attempts to seamlessly handover communication to achieve better performance. A node can be found in one of five states: Sleep, Receive, Send, Discover, and Handover. In the initial step, a node stays in Sleep state. The node wakes up at any time if it has data to send and enters the Send state. To receive data, the node periodically wakes up and changes its state to Receive. If the node detects a discovery request, it enters a Discovery state and searches for an intermediate neighbor before breaking the link. If the node receives a discovery reply, it enters Handover state. If the handover attempt is successful, the node shifts to Send state, or otherwise, goes back to sleep. A handover process is shown in Figure 3. When a sender recognizes the mobility of *Receiver 1* by estimating the RSSI of incoming ACK, the sender broadcasts data with a discovery request. *Receiver 2* and *Receiver 3* wake up to participate in discovering a new intermediate node that takes over *Receiver 1*. If *Sender 1* receives a discovery reply from *Receiver 2* or *Receiver 3*, *Sender 1* updates its routing configuration and sends the data for R1 to the intermediate node.

A new low duty cycle, energy efficient, mobility based protocol was introduced (Boarder Node MAC or BN-MAC) [22]. The hybrid approach of BN-MAC uses both contention and schedule based communication. A Broader Node (BN) is kind of a sink node that gathers information about the target region and communicates with other broader nodes as necessary. BN-MAC tries to guarantee end-to-end (source node to BN) reliability. Topology changes do not affect the performance of BN-MAC because all nodes maintain the information of only one-hop neighbor nodes that rarely changes. During end-to-end data transmission, BN-MAC tries to reduce the number of same data retransmission by sender nodes using an automatic packet buffering process. Therefore, intra-cluster communication between BN and member nodes is managed with an asynchronous method and inter-cluster communication among BNs is managed with a synchronous method. Our mechanism also considers the mobility and trade-off between energy efficiency and data transmission latency. However, the main difference between our mechanism and previous studies is that the proposed mechanism adaptively adjusts the node’s active period as its mobility. If a node quickly moves, its active period increases to track its location.

## 3. Mobility-Aware Adaptive Duty Cycling Mechanism in Tunnel Excavation

### 3.1. Tracking Objects

As the tunnel excavation progresses, the excavation face, i.e. blind end, gets farther away from the tunnel entrance. Accordingly, the equipment and construction workers have to move to the face to work; so their traveling distance gets longer. As previously mentioned in the introduction, a variety of equipment is required, depending upon the construction process. During the intermission between two consecutive processes, there is traffic to switch the equipment inside the tunnel being constructed, as well as construction workers. Even during a single construction process, traffic can also occur.

We designed a ZigBee [23] based system because Radio-Frequency Identification (RFID), that is typically applied technology to the tracking system [24], needs numerous anchors that are used for estimating the location of mobile objects and can only transmit limited information. The proposed system can conduct tracking of mobile objects by cooperation between a small number of anchors (cluster heads) with sensor nodes attached to mobile objects. Also, ZigBee based approach can offer support for a wide range of services by applying several sensors located in the tunnel environment.

**Figure 4:**
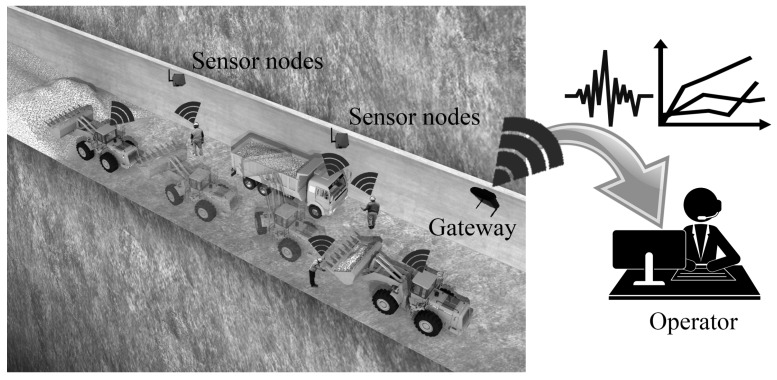
Illustration of tunnel excavation and tracking objects.

Figure 4 illustrates the tunnel excavation and tracking objects. In terms of wireless communication, the equipment and construction workers have attached monitoring devices (sensor nodes) that are equipped with a processor, sensors, and radio module in order to report their state to a remote operator as shown. Also various sensors, including inertial sensors such as accelerometer or gyroscope, are used to estimate positions and velocity of moving objects as well as detect dangerous situations at a tunnel excavation site. 

It is impossible to directly send data of each construction unit to the remote operator, so that sensor nodes compose groups (clusters) and send their data to a cluster head of each cluster. The cluster head gathers data of all member nodes and sends the collected data to the gateway that is installed at the entrance of the tunnel. Each cluster head connects to neighbor cluster heads, making up the backbone link, to send all kinds of monitoring data toward the gateway. The gateway can be connected to operator by wireless interfaces such as ZigBee, Bluetooth, WIFI, and Cellular (3G/LTE), which enables monitoring of further data. 

### 3.2. Network Model 

**Figure 5:**
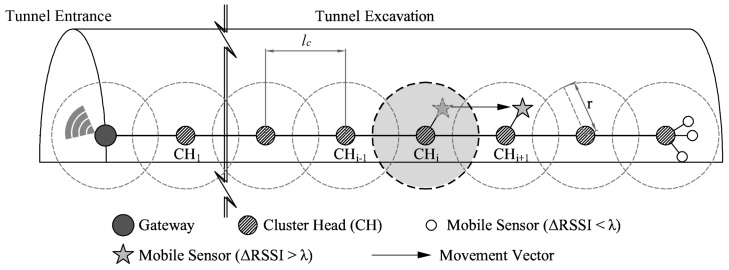
System model for tracking objects in tunnel excavation.

In clustering based protocols, for example LEACH [25], nodes make groups with their neighbors and direct the data of member nodes to a leader node called the cluster head, to reduce the amount of data that must be transmitted to the base station. This is because it is impossible and inefficient for all sensor nodes to communicate with the base station directly. Cluster heads near the base station consume more energy than other cluster heads due to a bottleneck in the data traffic. This problem can be solved by using advanced routing protocols [26,27]. Each cluster head (*CH_i_*) is connected with two neighbor cluster heads (*CH_i_*_−1_, *CH_i_*_+1_) and gathers data of the mobile sensors (member nodes) within its communication range *r,* as shown in Figure 5. If the mobile sensor changes its location as marked by the movement vector *v* and cannot communicate with the old cluster head (*CH_i_*), a new cluster head (*CH_i_*_+1_) that can communicate with the mobile sensor enrolls and manages the mobile sensor. In terms of mobile sensors, their movement determines their sleep time. Each node is defined as in a non-mobility state if and only if its change of RSSI value (ΔRSSI) is less than the threshold λ; whereas the mobility state implies the opposite. A sensor in a mobility state (ΔRSSI> λ) decreases its sleep time to frequently send its state information to the cluster head; whereas, a mobile sensor in a non-mobility state (ΔRSSI< λ), increases its sleep time to rarely send its state information to the cluster head. For example, as shown in Figure 4, the dump truck stopped to load mucks corresponds to the latter, and the pay loader that travels back and forth between muck and the dump truck corresponds to the former.

The distance between adjacent cluster heads *l_c_* must be less than or equal to *2r* to localize and track from at least one cluster head. In order to find a more appropriate range of *l_c_*, the minimum number of wake-up times within a certain cluster head n and the maximum velocity of the mobile sensors $v_{max}$ are required in sensor field [28]. Note that the user can choose a number n (≥ 1) depending on the tracking reliability; a higher n gives higher delivery performance for tracking. Thus, the time to travel $l_{c}$ with $v_{max}$ should be greater than or equal to (T+τ)·n as shown in Equation (1):

(1) $$\left(T+\tau \right)\cdot n\leq \frac{l_{c}}{v_{max}}$$

where T and τ is wake-up and sleep time in duty cycling interval, respectively. That is, *l_c_* should satisfy Equation (2):

(2) $$\left(T+\tau \right)\cdot n\cdot v_{max}\leq l_{c}\leq 2\cdot r$$

### 3.3. Energy Saving Strategy

**Figure 6:**
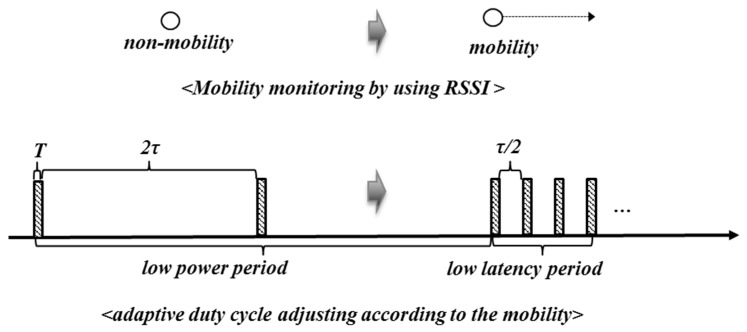
Mobility-aware adaptive duty cycling strategy (α=2, β=2).

In tunnel excavation, there are many mobile objects equipped with sensor nodes, such as equipment and workers. Each node sleeps most of the time (τ) to conserve energy consumption, periodically wakes up for a very short duration (*T*), and checks the medium for radio activity. For effective tracking in both static and mobile scenarios, we present a mobility-aware adaptive duty cycling mechanism, as shown in Figure 6. Each node is defined as in a non-mobility state if and only if its change of RSSI value (ΔRSSI) is less than the threshold λ; whereas the mobility state implies the opposite. Normally, this is designed to save energy of the static sensor nodes. In order to reduce the latency on high mobility while still maintaining a low power design, it adjusts its duty cycle ratio by decreasing or increasing the sleep time, depending on its mobility state as expressed in Equation (3):

(3) $$f_{\tau }\left(\alpha ,\beta \right)=\{\begin{array}{c} \begin{array}{c} \frac{\tau }{\alpha }\;\mathrm{if}\;\Delta RSSI\geq \lambda \\ \;\beta \tau \;\mathrm{otherwise} \end{array} \end{array}$$

where,$\;f_{\tau }\left(\alpha ,\beta \right)$ is the sleep time adjusting function, α and β are the factors that are greater than or equal to ‘1’. When α=1 and β=1 are employed, the duty cycle is equivalent to the periodic duty cycle mechanism. Note that these values can be manipulated depending upon the conditions: whether consuming energy reduction is more important or latency reduction is more important.

## 4. Analysis and Evaluation

In this section, we analyze the energy consumption and latency of our mobility-aware adaptive duty cycling mechanism for tracking objects during tunnel excavation. We evaluate the performance by comparing it with the periodic duty cycling mechanism under various mobility conditions. Table 1 lists the notations used in this paper.

### 4.1. Energy Consumption

We analyzed the energy consumption per unit time of both periodic and adaptive duty cycling mechanisms. The energy consumption per unit time is proportional to the ratio of wake-up time to duty cycle (*D*). For the simplest model, we assumed that the energy consumed for idle listening is approximately the same as the energy consumed for receiving and transmission in WSNs. Let $P_{W}$ denote the power for idle listening, receiving, and transmission. In the periodic duty cycling mechanism, the consumed energy, $\varepsilon_{Periodic}\;$, can be computed as:

(4) $$\varepsilon_{Periodic}\;=P_{W}\;\cdot D\;=\;P_{W}\;\cdot \frac{T}{T+\tau }\;$$

where D=T/(T+τ). Note that in the periodic duty cycling mechanism, a mobile sensor does not adjust its duty cycle, so the sleep time is not adjusted.

With the adaptive duty cycling mechanism, each mobile sensor can adjust its duty cycle by decreasing or increasing the sleep time, depending on whether its change of RSSI value (ΔRSSI) exceeds a certain threshold λ or not. For example, when α=2 and β=2 are employed, the duty cycle is adjusted by doubling or halving the sleep time. When the mobile sensors exceed the threshold with probability, *p*, the energy consumed per unit time for the adaptive duty cycling mechanism, $\varepsilon_{Adaptive}$, can be derived as:

(5) $$\begin{array}{ccc} \;\varepsilon_{Adaptive}\; & = & P_{W}\cdot \frac{T}{T+\frac{\tau }{\alpha }}\cdot p+P_{W}\cdot \frac{T}{T+\beta \tau }\;\left(1-p\right)\\ & = & P_{W}\cdot \frac{D}{D+\frac{1-D}{\alpha }}\cdot p+P_{W}\cdot \frac{D}{D+\beta \left(1-D\right)}\;\left(1-p\right)\; \end{array}$$

**Figure 7:**
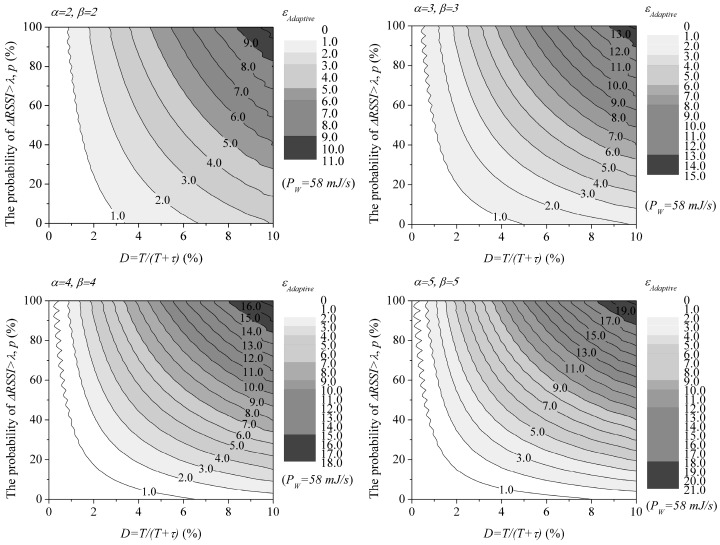
Examples of the contour of the energy consumed per unit time for the adaptive duty cycling mechanism.

Figure 7 shows a few examples of the contour of the consume energy per unit time for the adaptive duty cycling mechanism with various values of α and β when *P_W_* = 58 mJ/s. The energy consumption parameter is selected based on the CC2420 RF module [29]. As shown in the figure, the energy consumed increases with *p*. This is natural because the value of *p* should increase as the mobility of the sensor node increases, and this causes decrease of the sleep time. Accordingly, the energy consumption increases. This means that we cannot guarantee that the mobility-aware adaptive duty cycling mechanism always consumes less energy than the periodic one does. 

The mobility-aware adaptive duty cycling mechanism consumes less energy than the periodic one if and only if the following inequality $\varepsilon_{Periodic}\;\geq \;\varepsilon_{Adaptive}$ is satisfied. This inequality can be expressed in terms of p and *D* as below, using Equations (4) and (5):

(6) $$p\;\leq \;\frac{\left(\alpha D+1-D\right)\left(\beta -1\right)}{\alpha \beta -1}\;$$

**Figure 8:**
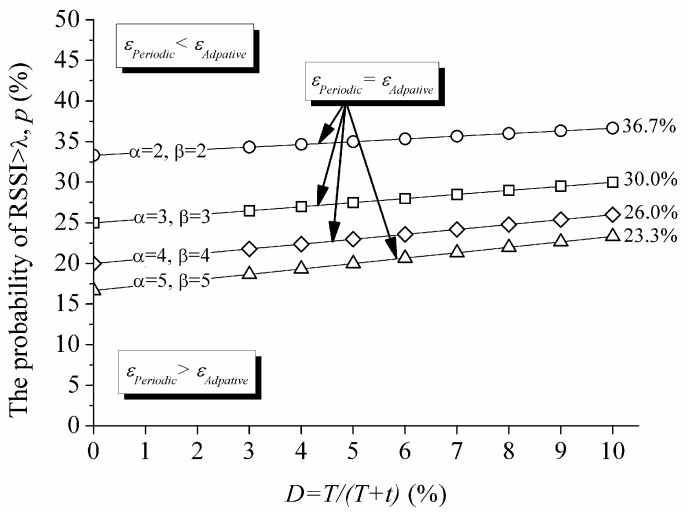
Comparison of energy consumption per unit time.

Figure 8 shows the comparison of energy consumption per unit time with the given values of α and β. For example, when α=2 and β=2 are employed and *D* is set smaller than 10% to save energy, approximately 36.7% probability is the upper limit that the mobility-aware duty cycle mechanism consumes less energy than the periodic one.

### 4.2. Expected Latency

We analyzed the expected latency for a packet transmission between two sensor nodes within the transmission range of one another. Because the latency is governed by the delay in sleep mode, we did not consider the processing, transmission, and propagation delays. We focused on the sleep latency during duty cycling mechanism. We first analyzed the latency of the periodic duty cycle mechanism with a fixed interval. Then, we evaluated our adaptive duty cycle mechanism by analyzing its ability to adapt to its mobility variations and compare it with periodic one.

For the periodic duty cycling mechanism, if a sensory event arrives at wakeup time [0, T], the sensor sends the data to the cluster head node without delay. However, during the sleep time [T, T+τ], the data will be sent in the next wakeup time. To be precise, let x be a random variable between [0, T+τ] denoting the data arrival time and let f(x) be the probability density function of x. As previously noted, τ is not adjusted for the periodic duty cycling mechanism. By substituting 1/(T+τ) for f(x), we obtain the expected latency of the periodic duty cycling mechanism:

(7) $$\begin{array}{ccc} L_{Periodic} & = & \int_{T}^{T+\tau }\left(T+\tau -x\right)f\left(x\right)dx\\ & = & \frac{1}{T+\tau }\int_{T}^{T+\tau }\left(T+\tau -x\right)dx\\ & = & \frac{\tau^{2}}{2\left(T+\tau \right)}\; \end{array}$$

For the adaptive duty cycling mechanism, we employed two adjusted sleep times, τ/α and βτ, according to change of the RSSI value. As described above, the latency for the adaptive duty cycling mechanism can be simplified to Equation (8):

(8) $$\begin{array}{ccc} \;L_{Adaptive}\; & = & \frac{{\left(\frac{\tau }{\alpha }\right)}^{2}}{2\left(T+\frac{\tau }{\alpha }\right)}\cdot p+\frac{{\left(\beta \tau \right)}^{2}}{2\left(T+\beta \tau \right)}\cdot \left(1-p\right)\\ & = & \left(\frac{\tau }{\alpha }-\beta \tau \right)\cdot \frac{\left\{T\left(\frac{\tau }{\alpha }+\beta \tau \right)+\frac{\beta }{\alpha }\tau^{2}\right\}}{2\left(T+\frac{\tau }{\alpha }\right)\left(T+\beta \tau \right)}\cdot p+\frac{{\left(\beta \tau \right)}^{2}}{2\left(T+\beta \tau \right)}\; \end{array}$$

**Figure 9:**
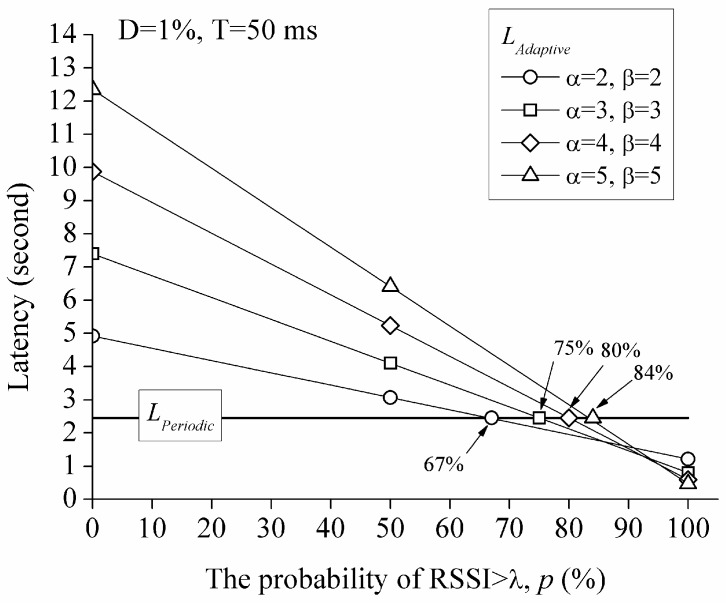
Comparison of $L_{Periodic}$ and $L_{Adaptive}$ when D=1% and T=50 ms.

Figure 9 shows the comparison between $L_{Periodic}$ and $L_{Adaptive}$ when D=1% and T=50 ms with various α and β. When α and β are given, $L_{Adaptive}$ is a linear function of *p* and decreases with increasing *p* owing to the negative coefficient of *p* as shown in Equation (8). In other words, $L_{Adaptive}$ decreases with increasing movement. This is natural because the value of *p* should increase as the mobility of the sensor node increases, and this reduces the sleep time.

As shown in Figure 9, by using various values of the sleep time adjusting factors, α and β, an engineer can manipulate the latency that has an advantage over the periodic mechanism. For example, when *p* is greater than 67%, $L_{Adaptive}$ with α=2 and β=2 has an advantage over $L_{Periodic}$.

### 4.3. Evaluation in Tunnel Excavation

To compare the proposed mobility-aware adaptive duty cycling mechanism and the periodic one, we simulated a dump truck that transfers excavated material out of the tunnel. During the mucking process, an empty dump truck goes into the tunnel, approaches the payloader working at the blind side, stops for loading, and then comes out of the tunnel. The dump truck typically moves inside the tunnel excavation site at the speed of 30 km/h, and it takes about 10 minutes to load a truck.

For the simulation analysis, we used α=2, β=2, *D* = 1%, *T* = 50 ms, and *P_W_* = 58 mJ/s. For evaluation of the proposed adaptive duty cycling mechanism, it was assumed that the condition of ΔRSSI>λ is always satisfied while the truck is moving, and the condition of ΔRSSI<λ is always satisfied while the truck is stopped. Accordingly, the sleep time is halved while the truck is moving, and is doubled while the truck is stopped for the loading. For the periodic mechanism, the sleep time is not adjusted.

**Figure 10:**
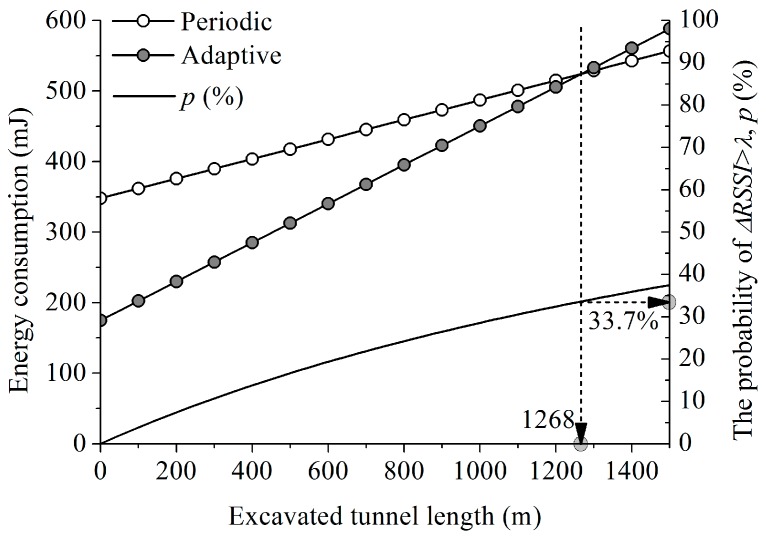
Energy consumption and the probability of ΔRSSI>λ with excavated tunnel length.

Figure 10 shows the energy consumed by the sensor tagged on the truck as the length of the tunnel excavation increases. With the given analysis condition, the proposed mobility-aware adaptive duty cycling mechanism consumes less energy than that of the periodic, until the length of the excavated tunnel reaches at 1268 m, in which case the probability is 33.7%. This probability is computed by dividing the truck moving time by the total staying time in the tunnel and corresponds to the value obtained from Equation (6). For the tunnel length$\;L_{Tunnel}$, the probability that the proposed mechanism consumes less energy than the periodic one can be computed as:

(9) $$\begin{array}{ccc} p=\;\frac{T_{M}}{T_{M}+T_{S}} & \leq & \frac{\left(\alpha D+1-D\right)\left(\beta -1\right)}{\alpha \beta -1}\\ \frac{2\cdot \frac{L_{Tunnel}}{v_{M}}}{2\cdot \frac{L_{Tunnel}}{v_{M}}+T_{S}} & \leq & \frac{\left(\alpha D+1-D\right)\left(\beta -1\right)}{\alpha \beta -1}\\ L_{Tunnel} & \leq & \frac{v_{M}\cdot T_{S}\cdot \left(\alpha D+1-D\right)\left(\beta -1\right)}{2\left\{\alpha \beta -1-\left(\alpha D+1-D\right)\left(\beta -1\right)\right\}}\; \end{array}$$

where $T_{M}$ is the duration of the truck moving, $T_{S}$ is the stopped time and $v_{M}$ is the velocity of the moving truck. Note that the excavation face is getting farther away from the tunnel entrance as the tunnel excavation progressed, so the travel distance of the dump truck gets longer. Accordingly, for the proposed adaptive mechanism, the number of duty cycles that use τ/α increases with the excavated tunnel length. As a result, the energy consumption increases faster than for the periodic case, which uses τ.

The latency of the two mechanisms was also analyzed with the given analysis conditions. The latency of the proposed adaptive mechanism was 1.23 s and 4.90 s while the truck is moving and stopped for the loading, respectively. For the periodic mechanism, the latency is 2.45 s regardless of the condition of truck movement. Recall that the purpose of this study is to enhance the safety at a tunnel excavation site by tracking moving objects. Considering this, the proposed adaptive duty cycling mechanism provides an advantage over the periodic mechanism and also consumes less energy than the periodic one does. Besides, if the tunnel is excavated from both ends, which is typical, the proposed mechanism can be applied to tunnels with length of about 2.5 km. 

The trade-off between reduction in energy consumed and reduction of latency is a fundamental issue in the design of duty cycling. The proposed mobility-aware duty cycling mechanism is also not free from this issue. By using various values of the sleep time adjusting factors, α and β, an engineer can manipulate the energy consumption per unit time or the latency that meets the specific requirements for a particular tunnel construction site.

## 5. Conclusions

Tunnel construction workers face dangers working in dark and restricted conditions. For safe tunneling, tracking mobile objects with low latency is critical to manage dangerous construction environments and to enhance safety during tunnel construction. In this paper, we designed a model to track objects during tunnel excavation based on the mobility-aware adaptive duty cycling mechanism. This mechanism employs an explicit strategy that maximizes the quality of tracking using high duty cycles when an object moves. To reduce energy consumption, the proposed mechanism minimizes the duty cycle by increasing the sleep time when the object is immobile.

We have compared the proposed mechanism to the periodic duty cycle mechanism by analyzing the expected energy consumption and the latency. In the investigated case, both the sleep time adjusting factors of α and β were employed, so the latency was adjusted by increase or decrease the sleep time, depending on the mobility condition of the node. The proposed mobility-aware adaptive duty cycling mechanism consumes less energy than the periodic duty cycling mechanism when Equation (6) is satisfied. In the simulated case of the dump truck tracking during the mucking process, the tunnel length for which the proposed mechanism would consume less energy than the periodic one was computed using the parameters of duty cycle, truck moving speed, and the time stopped.

## Figures and Tables

**Table 1:** Notation.

Notation	Description
ΔRSSI	The change of RSSI value
p	The probability that the ΔRSSI of sensor nodes exceeds a threshold λ
T	The wake-up time of duty cycle interval
τ	The sleep time of duty cycle interval
*D*	The duty cycle (i.e.,) D=T/(T+τ)
$P_{W}$	The power for wake-up
$\varepsilon_{Periodic}$	The expected energy consumption of periodic duty cycling mechanism with an fixed sleep time of τ.
$\varepsilon_{Adaptive}$	The expected energy consumption of adaptive duty cycling mechanism with two adjusted sleep time, τ/α and βτ, according to its change of RSSI value.
$L_{Periodic}$	The expected latency of periodic duty cycling mechanism with an fixed sleep time of τ.
$L_{Adaptive}$	The expected latency of adaptive duty cycling mechanism with two adjusted sleep time, τ/α and βτ, according to its change of RSSI value
